# Lactate Up-Regulates the Expression of Lactate Oxidation Complex-Related Genes in Left Ventricular Cardiac Tissue of Rats

**DOI:** 10.1371/journal.pone.0127843

**Published:** 2015-05-21

**Authors:** Daniele Gabriel-Costa, Telma Fatima da Cunha, Luiz Roberto Grassmann Bechara, Rodrigo Soares Fortunato, Luiz Henrique Marchesi Bozi, Marcele de Almeida Coelho, Maria Luiza Barreto-Chaves, Patricia Chakur Brum

**Affiliations:** 1 School of physical Education and Sport, University of São Paulo, São Paulo, Brazil; 2 Carlos Chagas Filho Institute of Biophysics, Federal University of Rio de Janeiro, Rio de Janeiro, Brazil; 3 Department of Anatomy, Institute of Biomedical Sciences, University of São Paulo, São Paulo, Brazil; Laurentian University, CANADA

## Abstract

**Background:**

Besides its role as a fuel source in intermediary metabolism, lactate has been considered a signaling molecule modulating lactate-sensitive genes involved in the regulation of skeletal muscle metabolism. Even though the flux of lactate is significantly high in the heart, its role on regulation of cardiac genes regulating lactate oxidation has not been clarified yet. We tested the hypothesis that lactate would increase cardiac levels of reactive oxygen species and up-regulate the expression of genes related to lactate oxidation complex.

**Methods/Principal Findings:**

Isolated hearts from male adult Wistar rats were perfused with control, lactate or acetate (20mM) added Krebs-Henseleit solution during 120 min in modified Langendorff apparatus. Reactive oxygen species (O_2_^●-^/H_2_O_2_) levels, and NADH and NADPH oxidase activities (in enriched microsomal or plasmatic membranes, respectively) were evaluated by fluorimetry while SOD and catalase activities were evaluated by spectrophotometry. mRNA levels of lactate oxidation complex and energetic enzymes MCT1, MCT4, HK, LDH, PDH, CS, PGC1α and COXIV were quantified by real time RT-PCR. Mitochondrial DNA levels were also evaluated. Hemodynamic parameters were acquired during the experiment. The key findings of this work were that lactate elevated cardiac NADH oxidase activity but not NADPH activity. This response was associated with increased cardiac O_2_^●-^/H_2_O_2_ levels and up-regulation of MCT1, MCT4, LDH and PGC1α with no changes in HK, PDH, CS, COXIV mRNA levels and mitochondrial DNA levels. Lactate increased NRF-2 nuclear expression and SOD activity probably as counter-regulatory responses to increased O_2_^●-^/H_2_O_2_.

**Conclusions:**

Our results provide evidence for lactate-induced up-regulation of lactate oxidation complex associated with increased NADH oxidase activity and cardiac O_2_^●-^/H_2_O_2_ driving to an anti-oxidant response. These results unveil lactate as an important signaling molecule regulating components of the lactate oxidation complex in cardiac muscle.

## Introduction

For many years, lactate was largely considered a dead-end metabolite and the major cause of skeletal muscle fatigue limiting exercise performance [1, 2]. However, this view was challenged by *in-vitro* and *in-vivo* studies demonstrating that lactate was not directly involved in muscle fatigue [3–5]. At present, the old lactate paradigm has shifted, and lactate is accepted as an important energetic intermediary, connecting metabolism of different organs and tissues, according to their energetic state [6–8]. Indeed, lactate can be released or up taken by skeletal muscles to be used as a fuel ultimately leading to ATP synthesis [7, 9]. The same is observed for the heart muscle, an important lactate consumer, especially under physiological stress, such as during physical exercise [10]. It has been demonstrated that lactate is also completely oxidized by cardiac myocytes after being up taken to generate energy for contraction and relaxation [8].

Lactate dehydrogenase (LDH) is a key mediator of lactate oxidation, being H4 the most prevalent LDH cardiac isoform empowering the formation of pyruvate from lactate [11]. This response will drive the lactate oxidation in mitochondrial matrix and intermembrane space, highlighting the importance of mitochondria to lactate-dependent energy production. Another role of LDH is the control of intracellular redox status. In fact, lactate-induced increase in reactive oxygen species (O_2_^●-^/H_2_O_2_) has been demonstrated in microsomal membranes, and is dependent of LDH/β-nicotinamide dinucleotide (NADH) oxidase. This is of particular interest since O_2_^●-^/H_2_O_2_ can act as second messengers driving the transcription of several genes. In fact, lactate modulates the transcription of genes involved in its own oxidation in skeletal muscle cells and this response is associated with increased hydrogen peroxide (H_2_O_2_) levels [12].

Considering that the flux of lactate in and out the heart is significantly high, we presently tested whether lactate perfusion of isolated rat hearts would up-regulate the expression of genes involved in the lactate oxidation. We further evaluated whether this response would be associated with specific lactate-induced increase in cardiac O_2_^●-^/H_2_O_2_ levels.

Here, we report that lactate perfusion of isolated hearts increased NADH oxidase activity, which was paralleled by enhanced O_2_^●-^/H_2_O_2_ production and superoxide dismutase (SOD) activity and that this response is specific for lactate and not to the metabolism of acetate. In line with our hypothesis, lactate-induced O_2_^●-^/H_2_O_2_ production was associated with an up-regulation of monocarboxylate transporter 1 (MCT1), monocarboxylate transporter 4 (MCT4), LDH and peroxissome proliferator receptor coativator type 1 alpha (PGC1α) (lactate oxidation complex). Altogether our findings highlight the importance of lactate as intracellular signaling molecule modulating physiological function in cardiac tissue.

## Materials and Methods

### Animals

Male Wistar rats weighing 250–300 g were used in this study. The animals were allocated into acrylic boxes lined with wood chips and access to water and food (rat chow) *ad libitum*. All procedures were in accordance to the *Guide for the Care and Use of Laboratory Animals (National Institute of health)* and approved by the *Ethics and Research Committee* of the University of São Paulo, SP, Brazil (#2011/55). The rats were instantaneously sacrificed by decapitation and their hearts were immediately removed and placed on Langendorff apparatus for isolated heart reperfusion preparation. The animals were divided in control and lactate (20 mM) or acetate (20 mM) groups.

### Isolated heart preparation

The isolated hearts were prepared as previously described by Vassalo et al. [13] with a few modifications. Briefly, the hearts transferred to the Langendorff apparatus were perfused through aorta under a constant flow (± 9 ml/min.) with Krebs-Henseleit (KH) solution, bubbled with a carbogenic gas mixture (95% O_2_ and 5% CO_2_), with pH ± 7.4 and temperature of 37°C. The hearts of the control group were perfused with filtrated (Swinnex filter holder: 47mm, membrane pore: 0.22 μm; EMD Millipore, MA, USA) KH solution of composition in (mM): NaCl 118.0; KCl 4.7; MgSO_4_ 1.66; KH_2_PO_4_ 1.18; CaCl_2_ 1.5; NaHCO_3_ 24.88; glucose 2.0. The hearts of the lactate and acetate groups were perfused with a modified filtrated KH solution, containing 20 mM of lactate (KH + Lactate 20 mM) and 20 mM of acetate (KH + Acetate 20 mM), respectively. These solutions were modified to maintain similar osmolarity of control solution/KH (KH: 316.4, KH + Lactate 20mM: 314.3 and KH + Acetate 20mM: 314.0 miliosmolar). KH + Lactate (20mM) and KH + Acetate 20 mM composition were in (mM): NaCl 97.0; KCl 4.7; MgSO_4_ 1.66; KH_2_PO_4_ 1.18; CaCl_2_ 1.5; NaHCO_3_ 24.88; glucose 2.0; Na-Lactate 20.0 or Na-Acetate 20.0. The concentration of 20 mM was chosen based on findings of Hashimoto et al. [12, 14, 15] who demonstrated that lactate significant changes O_2_^●-^/H_2_O_2_ production with this concentration in skeletal muscle, cardiac and liver tissues, respectively. All procedures, since decapitation until the beginning of the perfusion were conducted within 90 seconds. The left ventricle was perforated by puncture needle to avoid liquid accumulation. Thereafter, the left ventricle was accessed and a soft distensible balloon was placed in ventricular cavity through the mitral valve. The end diastolic pressure was settled to approximately 10 mmHg. The values of left ventricle developed pressure (DP), heart rate (HR), maximum positive and negative dP/dt (+dP/dt_max_ and-dP/dt_max_) and perfusion pressure (PP) were obtained by pressure transducers and subsequently amplified, digitalized and stored to further analysis (Power Lab-Lab Chart 7, AD Instruments, EUA).

### Experimental protocol

The hearts were equilibrated during 40 min after the introduction of the latex balloon beating spontaneously. After equilibration, the hearts were prefunded with KH or KH + Lactate (20 mM) or KH + Acetate (20 mM) solutions during 120 min. A time-course pilot experiment was conducted to evaluate the maintenance of contractile function and the responses of gene and protein expression after lactate perfusion. The peak responses were obtained at 120 min of perfusion (data not shown). Therefore, 120 min was chosen to demonstrate the effects of lactate in cardiac tissue. The perfusate was collected at 30, 60 and 120 min to evaluate pH and lactate concentrations. After 120 min the left ventricle was dissected and readily frozen in liquid nitrogen and then at -80°C.

### Determination of lactate concentration in the perfusate and cardiac homogenate

The determination of lactate concentration was based on Rosenberg and Rush [16] technique. Briefly, the tissues were treated with perchloric acid (3–6%, v/v) and homogenized and centrifuged by 20 min at 4°C at 10,000xg to reduce protein content of the samples. The supernatants and the perfusates were used to measure lactate concentration. To determine lactate levels, the samples were first incubated with 0.2 M of glycine-semycarbazide buffer and 0.02 M of NAD^+^ and the absorbance was measured in a wavelength of 340 nm (R_1_). Then, 2mg/ml of LDH obtained from rabbit muscle was added in the preparation and incubated for 60 min at 40°C. Thereafter, a second reading was obtained at the same wavelength (R_2_). The values were used to calculate the net absorbance with and without LDH, with the following formula A = (R_2_–0.9R_1_)–(B_2_–0.9B_1_), where A is the net absorbance, B_1_ is the blank absorbance without LDH and B_2_ is the blank absorbance with LDH. The concentration of lactate in mM was inferred by using the values of the net absorbance in a standard curve obtained between absorbance and increasing concentrations of lactate.

### Quantification of NADH and NADPH oxidase activities

The NADH oxidase activity and β-nicotinamide dinucleotide phosphate (NADPH) oxidase activity were obtained in both enriched microsomal membrane (EMM) and enriched plasmatic membrane (EPM) of perfused hearts, respectively.

These enriched fraction membranes were obtained as described by Fortunato, et al. [17]. Briefly, to obtain the EMM of cardiac tissue, the left ventricle was homogenized in phosphate sodium buffer containing in (mM): 50 sodium phosphate, 0.5 DTT (dithiothreitol), 1 EGTA (ethylene glycol-bis(2-aminoethylether)-*N*,*N*,*N′*,*N′*-tetraacetic acid), 250 sucrose, pH 7.2, 5 μg/ml aprotinin and 34.8 μg/ml PMSF (phenylmethanesulfonylfluoride) and centrifuged at 3,000xg, during 15 min at 4°C. Then, the supernatant was centrifuged at 100,000xg, during 30 min at 4°C and the pellet was resuspended in 1 ml of phosphate buffer. Another centrifugation was attained at 100,000xg during 30 min at 4°C. The pellet was then resuspended in 1 ml of phosphate buffer containing in (mM): 50 sodium phosphate, 1 EGTA, 2 MgCl_2_, 250 sucrose, 5 μg/ml aprotinin and 34.8 μg/ml PMSF, pH 7.2.

The EPM fraction was obtained by the centrifugation of the homogenized tissue at 3,000xg, during 15 min at 4°C and another subsequent centrifugation to obtain the pellet of the preparation. Both NADH and NADPH oxidases were measured in similar conditions. Briefly, the samples were incubated in a medium containing phosphate buffer (200 mM), pH 7.2, Amplex Red (50 μM), SOD (200 U/ml) and horseradish-peroxidase (0.25 U/ml). The reactions occurred with or without the corresponding substrates NADH or NADPH (0.1 mM, respectively). In order to calculate the specific activity of both enzymes, the slope of the curve with the addition of NADH/NADPH was subtracted from the slope of the curves with addition of water for each sample. The fluorescence was detected in a wavelength of 563nm to excitation and 587 to emission. A standard curve with known concentrations of H_2_O_2_ was used to transform the values to nmol H_2_O_2_/min/mg. As we used SOD in our preparations we referred O_2_^●-^/H_2_O_2_ as total H_2_O_2_ in figures.

### Dosage of reactive oxygen species

#### *Ex-vivo* analyses (Perfused hearts)

Lactate produces superoxide anion and H_2_O_2_ in bovine calf hearts and skeletal muscle lineage of rats [12, 14]. To measure the levels of O_2_^●-^/H_2_O_2_ in the heart, we used EMM since many oxidases, including NADH oxidase, are present in microsomal membranes of heart tissue. The EMM was used to access O_2_^●-^/H_2_O_2_ generation with incubation of 50 μM of Amplex Red, 200 U/ml of superoxide dismutase (SOD) and 0.25 U/ml of horseradish-peroxidase (HRP) during approximately 35 min at 30°C. The fluorescence was detected in a wavelength of 563 nm to excitation and 587 nm to emission. A standard curve with known concentrations of H_2_O_2_ was used to transform the values in μM of H_2_O_2_ from the maximal levels fluorescence (plateau) obtained curves (control and lactate perfused hearts). The same procedures were conducted with the EMM of the hearts perfused with acetate. As we used SOD in our preparations we referred O_2_^●-^/H_2_O_2_ as total H_2_O_2_ levels in figures.

#### *In-vitro* analyses (Non-perfused hearts)

To confirm the effect of lactate and acetate on O_2_^●-^/H_2_O_2_ generation an *in-vitro* assay was conducted with non-perfused hearts. The levels of O_2_^●-^/H_2_O_2_ were measured after addition of lactate (20 mM) or reduced NADH (1mM) or acetate (20 mM) directly in 96 well plates in the presence of EMM of the non-perfused hearts. The EMM of non-perfused hearts was obtained as previously described. The peak of the fluorescence was detected in a wavelength of 563 nm to excitation and 587 nm to emission in 96-wellplates. A standard curve was used to transform the maximal fluorescence values in μM of H_2_O_2_.

### Quantification of SOD and catalase

Superoxide dismutase (SOD) activity assay was based on the inhibition of xanthine/xanthine oxidase-driven cytochrome C reduction by SOD present in the sample. Left ventricles were homogenized in potassium phosphate buffer containing in (mM): 50 KH_2_PO_4_, 50 K_2_HPO_4_, pH 7.8 and centrifuged at 10,000xg for 20 min at 4°C. The supernatant was used to measure SOD activity. Initially, cytochrome C reduction rate was followed in absence of the sample for five minutes at absorbance 550nm in a mixture containing in (mM): 1.18 xanthine, 19 cytochrome C and xanthine oxidase, diluted in sodium phosphate buffer (50mM, pH 7.8). Xanthine oxidase concentration was adjusted in order to obtain a standard rate of cytochrome C reduction of 0.025 units of absorbance per minute. Following, cytochrome C reduction rate was measured in the presence of the sample (20μg) [18]. The difference between the two rates was attributed to SOD present in the tested sample. SOD activity was measured within the linear range of the assay.

Catalase activity was measured as described by Weydert and Cullen [19]. Muscle homogenates were obtained as above. The rate of H_2_O_2_ decomposition by catalase was assessed by following the decay in absorbance at 240nm for 4 min in the presence of 10mM H_2_O_2_.

### RNA extraction and quantitative real time RT-PCR

Total RNA was isolated from left ventricle samples using Trizol (Invitrogen, Carlsbad, California). The RNA concentration and purity (260:280 nm ratios) was assessed in a spectrophotometer (Nanodrop 2000, Thermo Scientific, Rockford, IL, USA) and integrity was observed in an agarose gel (2%, w/v) electrophoresis. The cDNA was synthesized using Revertaid First Strand cDNA synthesis kit (Fermentas, Glen Burnie, MD, USA. The genes analyzed were: MCT1, MCT4, Hexokinase (HK), LDH, Pyruvate dehydrogenase (PDH), Citrate synthase (CS), PGC1α, and cytochrome oxidase IV (COXIV), SOD1, SOD2, SOD3, Nuclear factor erythroid-2 related factor 1 (NRF-1), Nuclear factor erythroid-2 related factor 2 (NRF-2) and cyclophilin was used as a reference gene. All primers were synthesized by Fermentas (Fermentas, Glen Burnie, MD, USA) and their sequences are shown in Table 1. The amplifications were performed separately using MaximaHSYBR Green/ROX qPCR Master Mix (Fermentas, Glen Burnie, MD, USA) in ABI Prism 5700 Sequence Detection System (Applied Biosystems Inc, CA, USA). Results were expressed using the comparative cycle threshold (Ct) method as described by the manufacturer. The ΔCt obtained from the subtraction of target gene and the reference gene (cyclophylin) Ct’s was used to calculate de ΔΔCt from other groups in relation to control group. The expression values were calculated with 2^-ΔΔCt^. Control group levels were arbitrarily set to 1.

**Table 1:** qRT-PCR Primer sequences.

Gene	Forward	Reverse
MCT1	5’ACCGAGAGGGTCAGTGTTTG 3’	5’TGGAGGTAAGACTGCGTCAA 3’
MCT4	5’ GGTCCCCTGGCTGCTATTAT 3’	5’TCCCATGGTCACACAAAGAA 3’
HK	5’ CATGAGGAAGATGCTTGCCG 3’	5’ CTACCAGCACTCTGCTTGCC 3’
LDH	5’ GCAGCAGGGTTTCTATGGAG 3’	5’TGGAGACAGTGGGATTGTCA 3’
PDH	5’ CCGAGCTGAAGGATGACCAA 3’	5’ ATAATCCCGGGAGAGGCCAT 3’
CS	5’ AACTGGACAGATGCCCACAG 3’	5’ AGACATGGGGTGCAGATTGG 3’
COX (IV)	5’ GAACAAGGGCACCAATGAGT 3’	5’GTTGACCTTCATGTCCAGCA 3’
PGC1α	5’ GCGGACAGAACTGAGAGACC 3’	5’CCATCATCCCGCAGATTTAC 3’
SOD 1	5’ TGTGTCCATTGAAGATCGTGTG 3’	5’CTTCCAGCATTTCCAGTCTTTG 3’
SOD2	5’ GGACAAACCTGAGCCCTAAG 3’	5’CAAAAGACCCAAAGTCACGC 3’
SOD3	5’ GACCTGGAGATCTGGATGGA 3’	5’GTGGTTGGAGGTGTTCTGCT 3’
NRF-1	5’ GGCGCAGCACCTTTGGAGAATGTG 3’	5’CATCGATGGTGAGAGGGGGCAGTTC 3’
NRF-2	5’ GGCAGGAGCTATTTTCCATTCCCGAG 3’	5’CTGGGGACAGTGGTAGTCTCAGCCTGC 3’
Cyclophilin	5’ TGGCAAGCATGTGGTCTTTGGGAAG-3’	5’ GGTGATCTTCTTGCTGGTCTTGCCATTC-3’

### Measurement of mitochondrial DNA content

Genomic DNA was extracted using lysis buffer containing in (mM): 100 Tris-HCL, 5 EDTA (ethylenediamine tetraacetic acid), 200 NaCl and 0.2% (w/v) SDS (sodium dodecyl sulfate), pH 8.0 plus 0.2 ug/ul proteinase K. The concentrations of DNA were measured using a spectrophotometer (Nanodrop 2000, Thermo Scientific, Rockford, IL, USA). The amplification of DNA content was obtained using specific primers for mitochondrial encoded gene (COXII). A nuclear encoded gene (cyclophilin) was quantified and used as control. The amplification of the genes and the level of mitochondrial DNA were attained by real time PCR with similar conditions already described for real time RT-PCR.

### Quantification of nuclear NFR-2

In order to evaluate whether lactate would increase nuclear NRF-2 expression in response to increased O_2_^●-^/H_2_O_2_, an immunoblotting for NRF-2 was performed in nuclear fraction of control and lactate perfused (20 mM) hearts. The cellular fractioning was performed as described by Oliveira et al. [20]. Briefly, 50 mg of heart tissue was homogenized in 500 μl of ice-cold RIPA buffer containing in mM: 150 NaCl, 50 Tris-HCl and 0.5% (w/v) deoxycholate, 1% (v/v) Triton X-100, 1:300 sigma protease inhibitor cocktail (Sigma Aldrich, MO, USA) at pH 7.4. The homogenate was centrifuged at 100xg for 5 min at 4°C. The supernatant was centrifuged at 600xg for 5 min at 4°C. The pellet was incubated with RIPA buffer containing 0.3% (w/v) SDS and DNAse (1 mg.ml−1) for 30 min at 4°C and then centrifuged at 2,000xg for 10 min at 4°C to obtain the nuclear extract (supernatant). The nuclear content was prepared and resolved in 10% (w/v) SDS-PAGE. The proteins were transferred to nitro-cellulose membranes, blocked with 5% (w/v) of BSA (bovine serum albumin) for 60 min at room temperature. The membranes were incubated overnight at 4°C with the polyclonal rabbit Anti-NFR-2 diluted 1:1000 (Santa Cruz Biotechnology, CA, USA) and further incubated with horseradish-peroxidase conjugated antibody diluted 1:10.000. The signal antibody binding was detected using ECL-plus (Enhanced Chemiluminescence, Amersham Biosciences, SP, Brazil). Densitometry measurements were calculated with Image J (1.46).

### Quantification of metabolic enzyme activities

The enzymatic activities of HK, LDH, PDH and CS were determined in left ventricular tissue homogenates.

#### Hexokinase

Left ventricular tissues were homogenized in Tris-HCl buffer containing in (mM): 75 Tris-HCl, 7.5 MgCl_2_, 0.8 EDTA, 1.5 KCl and 4 mercapto-ethanol, pH 7.5 and centrifuged at 10,000xg for 20 min at 4°C. The supernatant was used to measure HK activity as described by Zammit and Newsholme [21]. The HK activity was obtained by monitoring the reduction of NADP^+^ via coupled reactions. The reduction of NADP^+^, on the other hand, was proportional to HK activity when measured at 340 nm. The working solution used in this assay was in (mM): 75 Tris-HCl, 7.5 MgCl_2_, 0.8 EDTA, 1.5 KCl, 4 mercapto-ethanol, 0.4 NADP^+^, 2.5 ATP, 1.4 units/ml of creatine phosphate, 0.05% (v/v) Triton X-100, excess G6PDH and 150 μg of protein. The reaction was started with 1 mM of glucose and was monitored during 20 min at 25°C at 340nm.

#### Lactate dehydrogenase

LDH was measured by an adaptation of the protocol described by Favero et al. [22]. Left ventricle tissues were homogenized in Tris-HCl buffer containing in (mM): 1000 Tris-HCl, 5 EDTA, pH 8.0. The supernatant was obtained as described above. The assay was based on monitoring the oxidation of NADH in the presence of pyruvate. The oxidation of NADH, on the other hand was proportional to LDH activity when measured at 340nm. The working solution used in this assay was in (mM): 75 Tris-HCl, 1000 EDTA, 2 NADH and 30 μg of protein. Sodium pyruvate (10 mM) was added after an incubation period of 10 min at 37°C and the reaction was monitored during 5 min at 340nm.

#### Pyruvate dehydrogenase

PDH was measured as proposed by Hinmam and Blass [23]. Briefly, the left ventricle tissues were homogenized in phosphate buffer containing in (mM): 50 potassium phosphate, 1 2-mercaptoethanol, 1 EDTA, and 0.1% (v/v) Triton X-100, pH 7.8. The supernatant was obtained as described above. The assay was based on the reduction of NAD^+^ by the oxidation of p- iodonitrotetrazolium violet (INT) dye. The amount of NAD^+^ oxidation was proportional to PDH activity when measured at 500 nm. The composition of the reaction mixture was in (mM): 0.6 INT, 2.5 NAD^+^, 0.2 thiamin pyrophosphate, 0.1 coenzyme A, 0.3 DTT, 1 MgCl_2_, and 0.0065 PMS, 1 mg/ml of BSA and 40 μg of protein. The absorbance was monitored after addition of 5 mM of sodium pyruvate during 25 min in 25°C at 500nm.

#### Citrate synthase

The CS activity was measured as described by Alp et al. [24]. Left ventricular tissues were homogenized with phosphate buffer containing in (mM): 50 sodium phosphate and 1 EDTA, pH 7.4. The supernatant was obtained as described above. The assay was based on the reaction of CoASH with dithiobis nitrobenzoic acid (DTNB) dye. The rate of CoASH and DTNB reaction was proportional to CS activity. The mixture containing in (mM): 100 Tris-base, 1 EDTA, 0.2 DTNB, 0.1 acetyl-coA, 1% (v/v) Triton X-100 and 0.5 oxaloacetate and 15 μg of protein was used in assay. The absorbance increase at 412nm was monitored during 7 min at 25°C.

### Statistical analyses

All data are expressed as means ± SE. The means were compared with non-paired t-test and were considered different when P < 0.05.

## Results

To test whether lactate administration would acidify the isolated heart perfusate, we have measured pH along 120 min of lactate perfusion at 20 mM. As expected, there was no acidification of the preparations as shown in Table 2, which suggests that lactate acidosis per se would not interfere in the results. We also verified lactate concentration in the perfusate and perfused hearts at 120 min. As depicted in Table 3, lactate perfusion significantly increased lactate concentration to physiological levels in perfused hearts and also increased perfusate levels.

**Table 2:** pH values of perfusate during 120 min of perfusion with KH or KHL.

	0 min	30 min	60 min	120 min
Control	7.7 ± 0.1	7.7 ± 0.1	7.6 ± 0.1	7.8 ± 0.0
Lactate	7.4 ± 0.0	7.6 ± 0.1	7.7 ± 0.0	7.6 ± 0.1

KH = Krebs-Henseleit and KHL Krebs-Henseleit + Lactate (20 mM); Values are mean ± SE of 5–7 hearts.

**Table 3:** Lactate concentrations on the left ventricle and perfusate after 120 min of reperfusion with KH or KHL.

Samples	[Lactate] Left Ventricle (mM)	[Lactate] Perfusate (mM)
Control	1.5 ± 1.4	3.8 ± 1.2
Lactate	4.8 ± 0.7*	21.2 ± 0.2*

KH = Krebs-Henseleit and Krebs-Henseleit + Lactate (20 mM); Values are mean ± SE of 5–7 hearts.

*indicates p<0.05 vs. control group.

### NADPH and NADH oxidase activities and O_2_^●-^/H_2_O_2_ production in lactate perfused hearts

**Fig 1:**
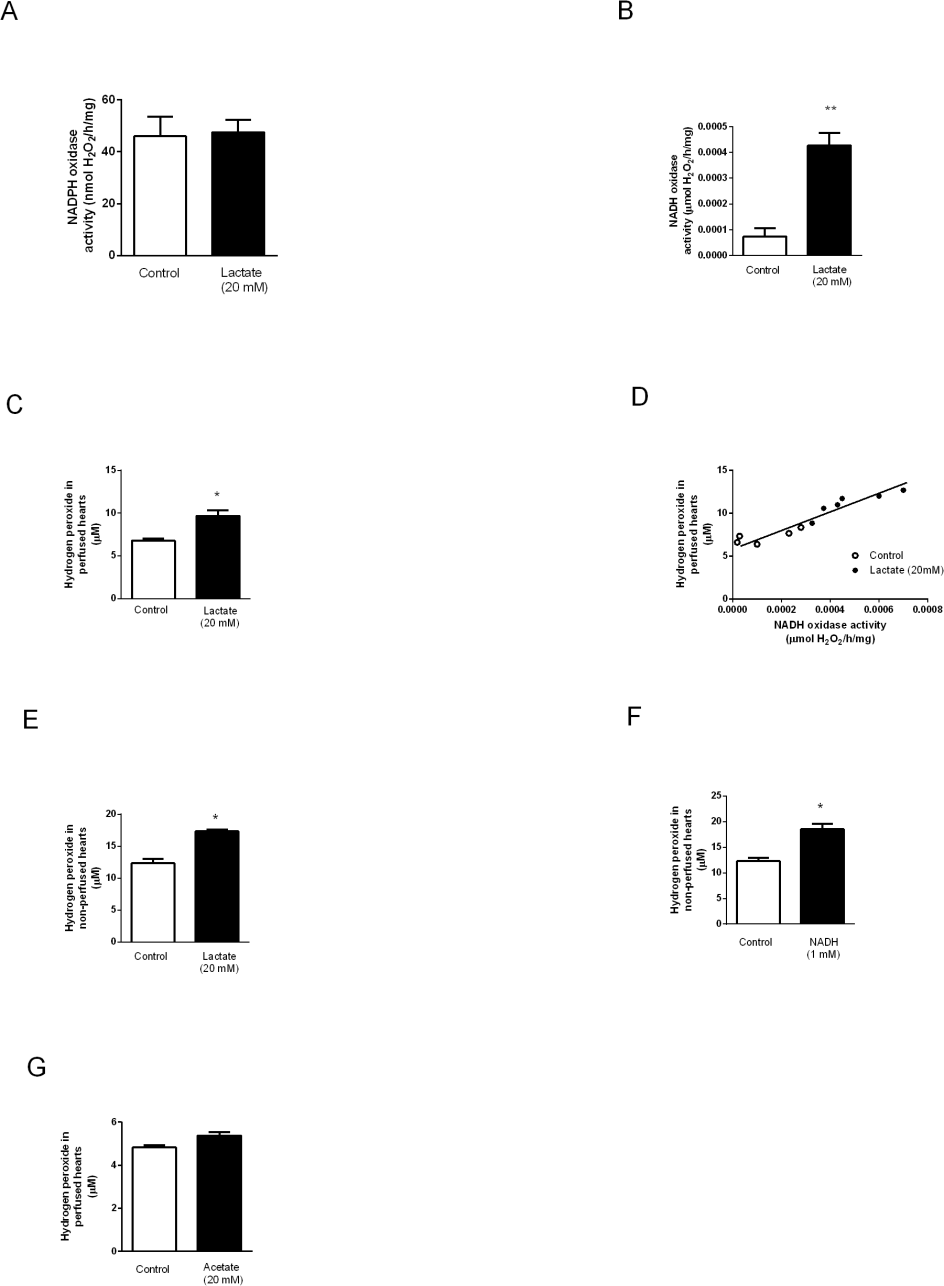
NADPH and NADH oxidase activities and reactive oxygen species (O_2_^●-^/H_2_O_2_) levels in perfused and non-perfused hearts. **(A)** NADPH oxidase, **(B)** NADH oxidase activities, **(C)** O_**2**_^**●-**^/H_**2**_O_**2**_ levels and **(D)** correlation between NADH oxidase activity and O_**2**_^**●-**^/H_**2**_O_**2**_ concentration in hearts perfused with KH or KH + lactate (20 mM) solutions during 120 min. Levels of O_**2**_^**●-**^/H_**2**_O_**2**_ in non-perfused hearts challenged with **(E)** lactate and **(F)** NADH. **(G)** Levels of O_**2**_^**●-**^/H_**2**_O_**2**_ concentration in perfused hearts challenged with acetate. Values are mean ± SE of 10 hearts; *indicates p<0.05 and **indicates p<0.01 vs. control group.

As lactate modulates superoxide anion production in cardiac myocytes [14], we have evaluated lactate-dependent NADH and NADPH oxidase activities in perfused hearts. No significant difference was observed in enriched membrane fraction NADPH oxidase activity of control and perfused hearts (Fig 1A). In contrast, NADH oxidase activity was significantly increased in EMM of lactate perfused hearts (Fig 1B). The increased NADH oxidase activity in hearts perfused with lactate was paralleled by significant O_2_^●-^/H_2_O_2_ production (Fig 1C). Interestingly, NADH oxidase activity and O_2_^●-^/H_2_O_2_ generation displayed a positive and significant correlation pattern (R^2^ = 0.90, p < 0.0001, Fig 1D). To further confirm that lactate and NADH oxidase were involved in O_2_^●-^/H_2_O_2_ production independently of perfusion condition, we pre-treated EMM of non-perfused hearts with lactate or NADH (substrate for NADH oxidase). Corroborating data obtained on the perfused hearts, both lactate (Fig 1E) and NADH (Fig 1F) significantly increased O_2_^●-^/H_2_O_2_ production in EMM. In order to test whether elevated levels of O_2_^●-^/H_2_O_2_ in lactate perfused hearts would be specific and not a byproduct of increased carbon metabolism, we have perfused hearts with acetate, another monocarboxylate. As shown in Fig 1G, no significant difference was detected in O_2_^●-^/H_2_O_2_ production in acetate perfused EMM confirming the lactate-dependent effect in heart´s EMM O_2_^●-^/H_2_O_2_ production.

### Antioxidant signaling activation

**Fig 2:**
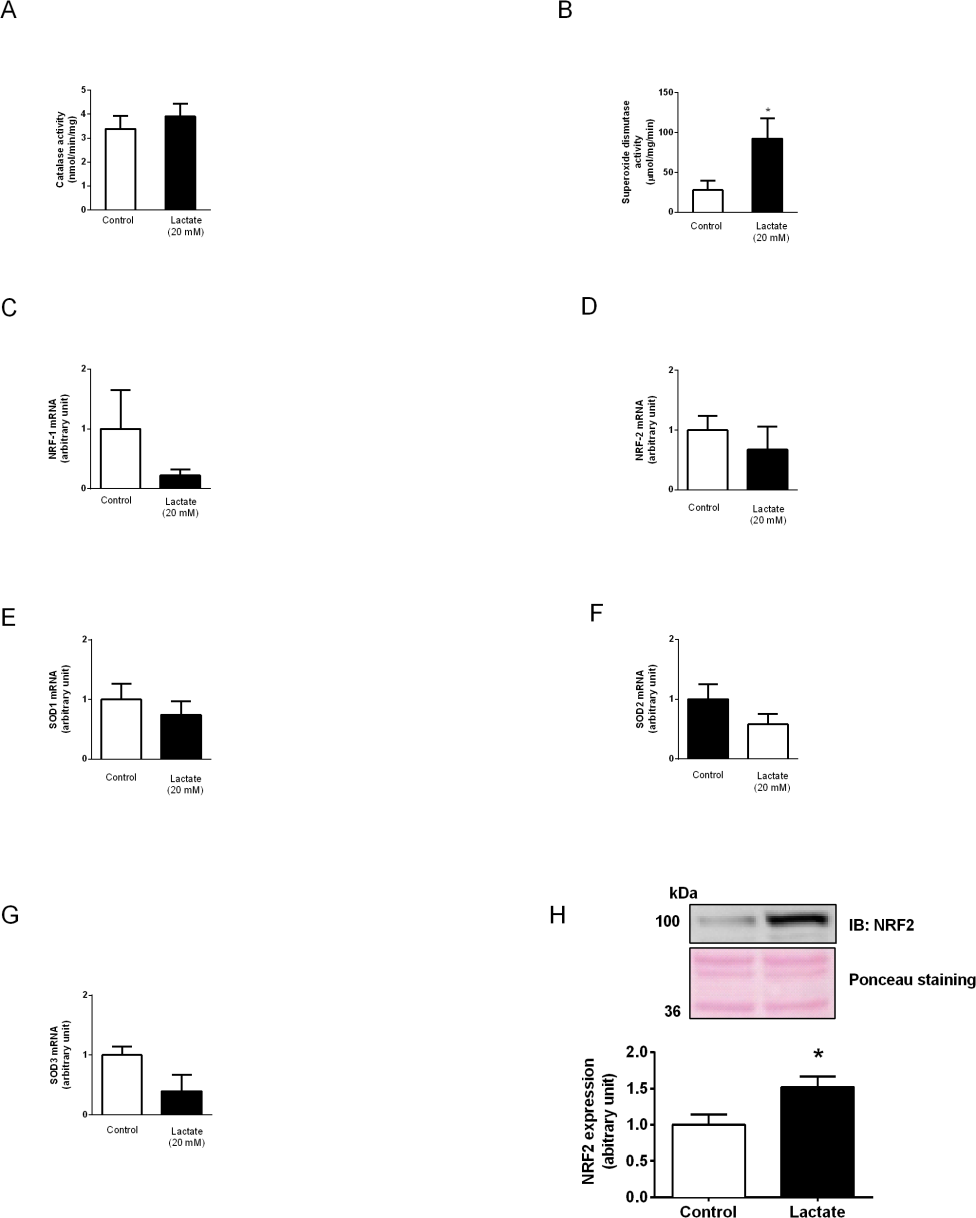
Activities of antioxidant enzymes and gene expression. Activities of (**A**) catalase and **(B)** SOD. mRNA levels of **(C)** NRF-1, **(D)** NRF-2, **(E)** SOD1, **(F)** SOD2 and **(G)** SOD3. **(H)** Nuclear NRF-2 expression in hearts perfused with KH or KH + lactate (20 mM) solutions during 120 min. Values are mean ± SE of 6–9 hearts; *indicates p<0.05 vs. control group.

To test whether increased levels of cardiac O_2_^●-^/H_2_O_2_ after lactate would activate antioxidant system defense, we have quantified the activity of the antioxidant enzymes, SOD and catalase. As shown in Fig 2A lactate perfusion had no impact on catalase activity. In contrast, SOD activity was significantly increased (Fig 2B). Although no difference was observed in NRF-1 (Fig 2C) and NRF-2 (Fig 2D) (transcription factors that up-regulate SOD gene expression) and SOD isoform mRNA levels (SOD 1, 2 and 3) in lactate perfused hearts (Fig 2E–2G). A significant increase in nuclear-NRF2 expression was observed in lactate perfused hearts (Fig 2H), which suggests that lactate might activate anti-oxidant response element (ARE) signaling cascades.

### Effect of lactate perfusion on energetic metabolic enzymes and lactate oxidation complex-related genes and enzymatic activities

**Fig 3:**
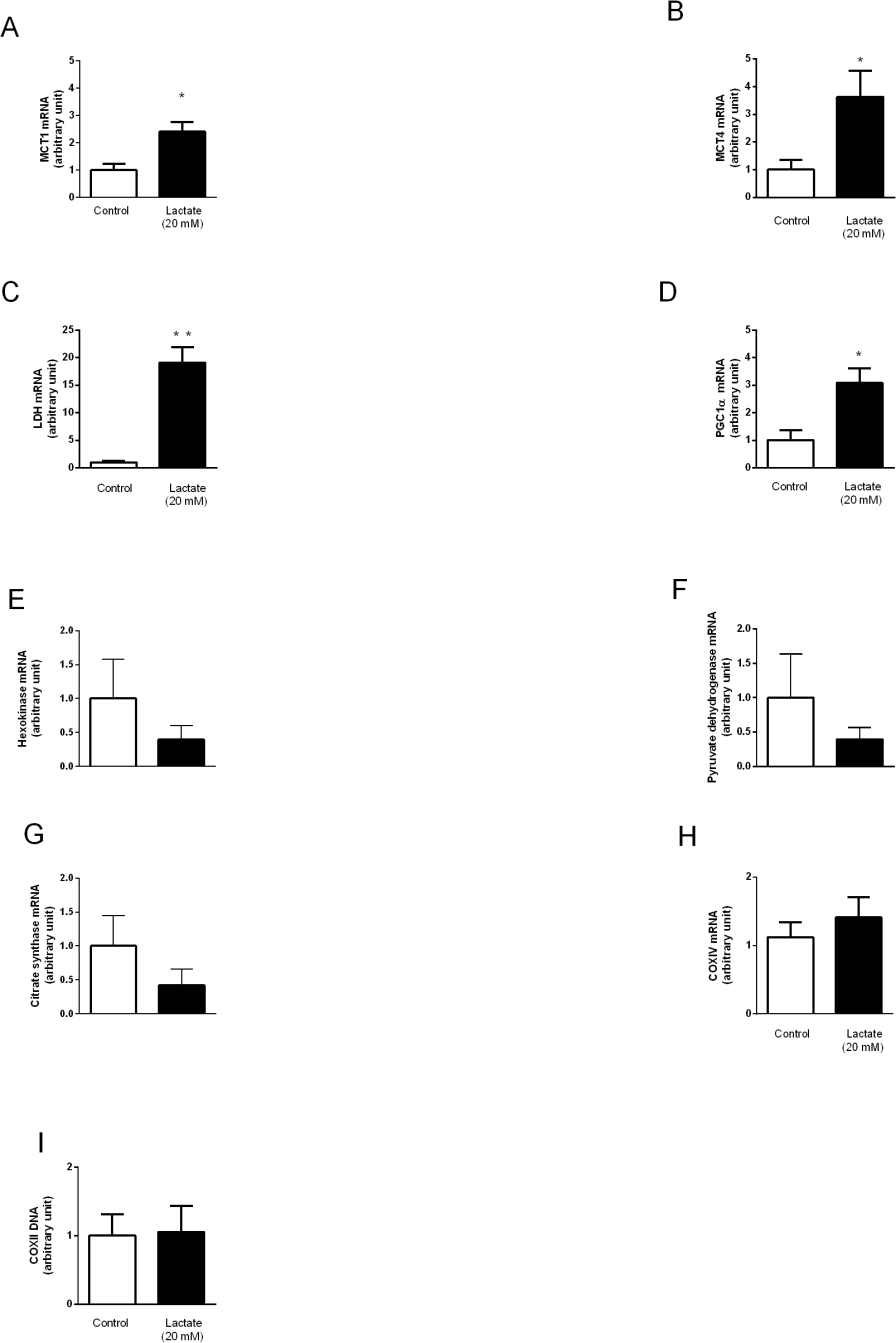
Lactate oxidation complex and energetic metabolic enzymes genes expression and mitochondrial DNA content. **(A)** MCT1, **(B)** MCT4, **(C)** LDH, **(D)** PGC1α, **(E)** HK, **(F)** PDH, **(G)** CS, **(H)** COXIV mRNA levels and **(I)** mtDNA level in hearts perfused with KH or KH + lactate (20 mM) solutions during 120 min. Values are mean ± SE of 6–9 hearts. *indicates p<0.05 and **indicates p<0.01 vs. control group.

**Fig 4:**
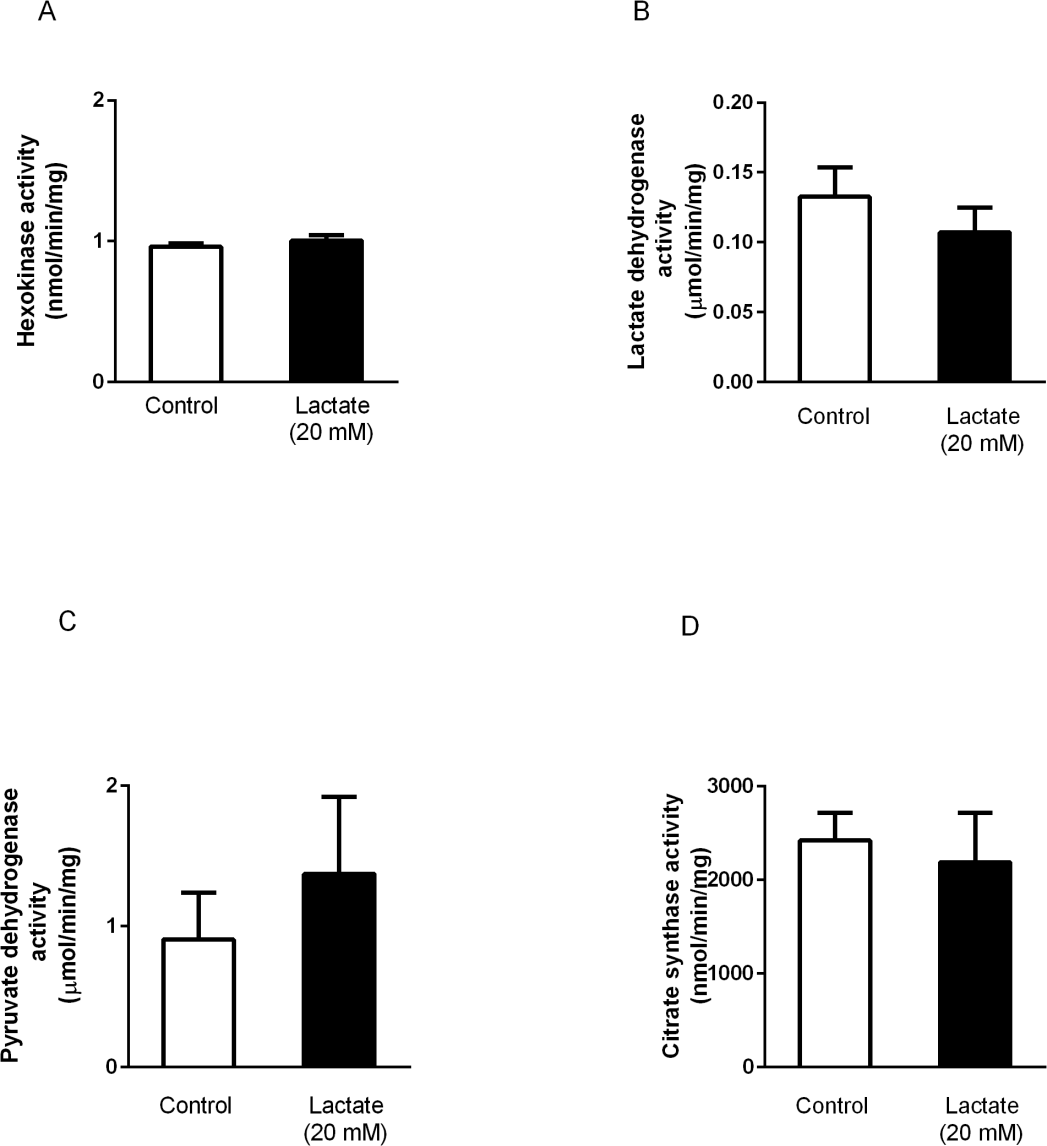
Activities of enzymes involved in lactate turnorver. Activities of **(A)** HK, **(B)** LDH, **(C)** PDH, and **(D)** CS in hearts perfused with KH or KH + lactate (20 mM) solutions during 120 min. Values are mean ± SE of 6 hearts.

Lactate perfusion significantly increased mRNA levels of MCT1 (Fig 3A), MCT4 (Fig 3B), LDH (Fig 3C) and PGC1α (Fig 3D). However, no changes were observed in mRNA levels of HK (Fig 3E), PDH (Fig 3F), CS (Fig 3G), COXIV (Fig 3H) or mitochondrial DNA copies (Fig 3I). We further analyzed the activity of enzymes involved in the regulation of lactate turnover. Fig 4 shows that no differences were observed between control and lactate perfused hearts on activity of HK (Fig 4A), LDH (Fig 4B), PDH (Fig 4C) and CS (Fig 4D).

### Effect of lactate on hemodynamic parameters

**Fig 5:**
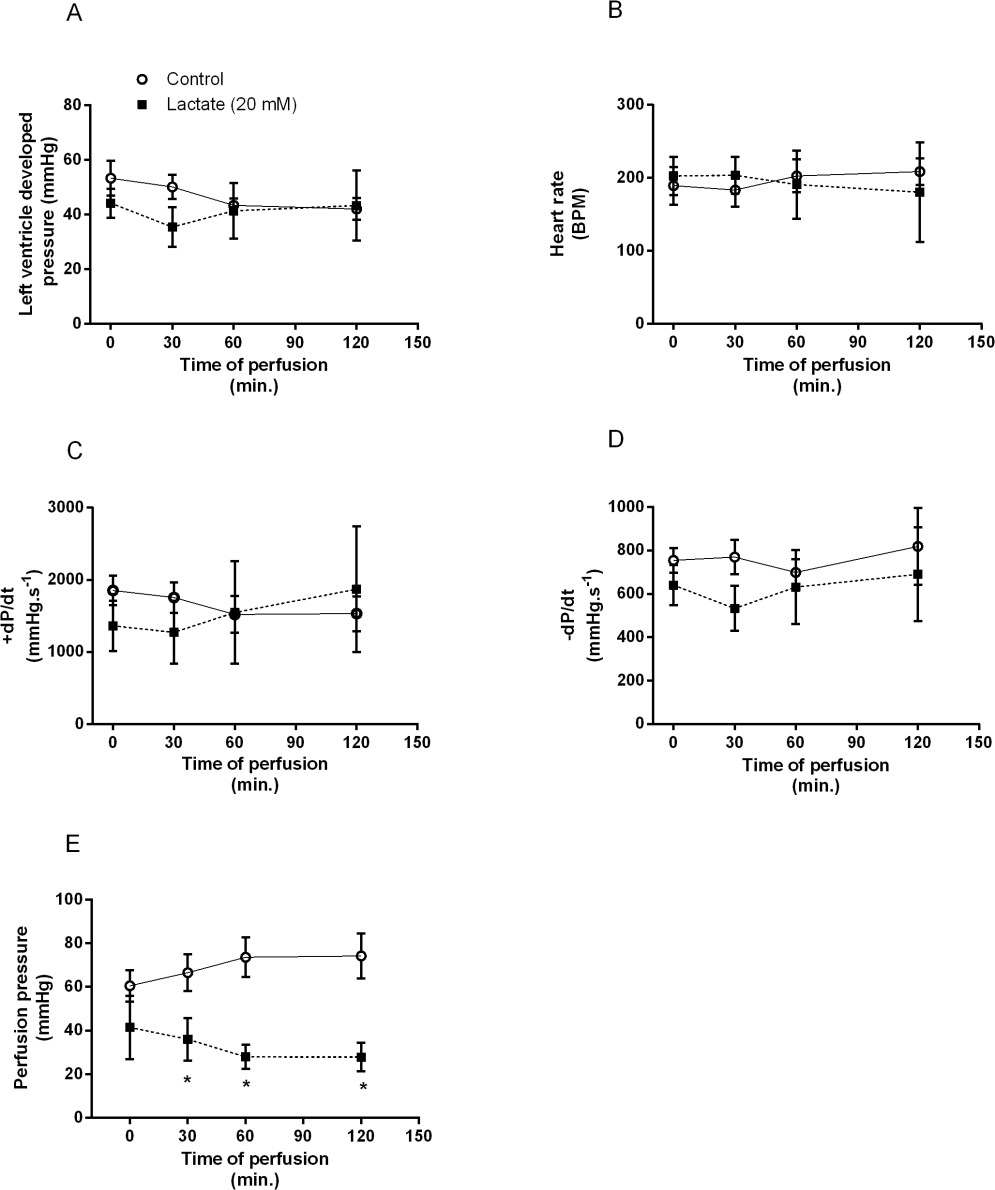
Hemodynamic parameters measured in perfused hearts. **(A)** Left ventricular developed pressure, **(B)** Heart rate, **(C)** +dP/dt_**max**_, **(D)**-dP/dt_**max**_ and **(E)** Perfusion pressure after 120 min of perfusion with KH or KH + lactate (20mM) solutions. Values are mean ± SE of 10–15 hearts; *indicates p<0.05 vs. control group.

In order to verify whether lactate perfusion would change isolated heart function, we have assessed hemodynamic variables. As noted in Fig 5, no significant differences were observed in left ventricle developed pressure (Fig 5A), heart rate (Fig 5B), +dP/dt (Fig 5C),-dP/dt (Fig 5D). However, lactate perfusion significantly reduced PP values from 30 to 120 minutes (Fig 5E).

## Discussion

This study demonstrates, for the first time, that lactate up-regulates cardiac genes involved in cellular lactate oxidation complex and this response is associated with an increased lactate-induced cardiac O_2_^●-^/H_2_O_2_ production.

For much of the 20^th^ century, lactate was largely considered a dead-end metabolite and the major trigger of acidosis-induced muscle fatigue [6]. However, at the present, lactate is accepted as a metabolic intermediary besides being an important fuel to the heart [25]. To further explore the role of lactate as a metabolic intermediary in cardiac tissue, we have set up a Langerdorff assay in which isolated hearts were perfused with exogenous lactate at 20mM concentration. Although one might suggest this would be a supraphysiological lactate concentration, this perfusate concentration induced a physiological increase of lactate concentration in cardiac tissue (~5mM) and no acidification of the perfusate (Table 2). These values suggest no interference of acidosis in our experiments.

As already demonstrated in different tissues (e.g. skeletal muscles, liver and heart), physiological increases in lactate induces local O_2_^●-^/H_2_O_2_ production [12, 14, 15], the latter an important intracellular messenger leading to signaling induction of numerous cellular responses. In fact, slight O_2_^●-^/H_2_O_2_ accumulation in skeletal muscle cell lines up-regulate redox-sensitive transcription factors, such as NRF-2 [26]. We presently demonstrate that lactate perfusion significantly increase the levels of cardiac O_2_^●-^/H_2_O_2_ confirming data from the literature. Of interest, increased lactate-induced O_2_^●-^/H_2_O_2_ production in cardiac tissue was associated with increased NADH oxidase but not NADPH oxidase activity. To further confirm the contribution of NADH to lactate-induced O_2_^●-^/H_2_O_2_ production, we challenged cardiac EMM membranes with lactate or NADH (substrate for NADH oxidase) and observed increased cardiac O_2_^●-^/H_2_O_2_ levels. We have also evaluated the lactate response-specificity challenging the hearts EMM with another monocarboxylate (acetate) and no difference was observed in O_2_^●-^/H_2_O_2_ levels. In the present study, we postulated that lactate would be able to rise O_2_^●-^/H_2_O_2_ levels through the action of the NADH oxidase and not by its oxidation in mitochondria. This is a system present in microsomal membranes that is dependent of LDH activity, which changes cell’s redox state (NAD^+^/NADH). Although mitochondria could be present in EMM fractions, they seem not to produce O_2_^●-^/H_2_O_2_ [27]. Instead, they could be an important source of LDH (H4) required to increase NADH and also NADH oxidase activity. In fact, it seems that lactate oxidation by mitochondria does not enhance O_2_^●-^/H_2_O_2_ levels [14, 28]. It also does not change oxygen consumption in isolated mitochondria [29] and tissue oxygen consumption rate [30]. Although our data provide evidence for increased O_2_^●-^/H_2_O_2_ via lactate-dependent NADH activity, we cannot exclude O_2_^●-^/H_2_O_2_ production via mitochondria in our perfused hearts.

In our study, the increased O_2_^●-^/H_2_O_2_ (∼10 μM) induced by lactate was paralleled by increased NRF-2 nuclear expression and up-regulation of genes driving lactate oxidation (MCT1, MCT4, LDH). These results corroborate O_2_^●-^/H_2_O_2_ to serve, mainly, as signaling molecules and avoiding them to exert direct toxic effects [31–33]. In fact, lactate increases O_2_^●-^/H_2_O_2_ by a redox mechanism in cells that are not under oxidative stress and this localized small production triggers several cellular responses [12, 14]. This is not case when oxidative stress occurs (40–80 μM of O_2_^●-^/H_2_O_2_) and cells need to be protected from oxidative damage. In this case, pyruvate, derived from lactate [34], can quench H_2_O_2_ and its anti-oxidant effect overrides lactate oxidation [35].

The net effect of O_2_^●-^/H_2_O_2_ on cells’ signaling pathways depends on the cellular antioxidant capacity. In fact, the antioxidant cellular network plays a primary role in maintaining O_2_^●-^/H_2_O_2_ within a physiologically compatible threshold level. In light of this, our findings of increased SOD but not catalase activity in lactate-perfused hearts confirm SOD contribution for redox homeostasis in physiological levels of lactate-induced cardiac O_2_^●-^/H_2_O_2_ accumulation. Of interest, the increased SOD activity was not accompanied by up-regulation of SOD and NRF isoform mRNA levels. However, the increase in NRF-2 nuclear expression corroborates the hypothesis that NRF-2 is translocating to the nucleus in response to lactate and NADH oxidase activity.

In line with our main hypothesis, lactate perfusion of isolated hearts up-regulated MCT1, MCT4, LDH and PGC1α mRNA levels, which play an important role in lactate oxidation and usage as energy fuel. Indeed, it has been proposed that lactate facilitates its own oxidation by enhancing its metabolic machinery [12, 36]. This is of particular interest since lactate becomes one of the most important fuels oxidized by the heart when its concentration is locally increased [36–39]. The fact that no changes were observed in the activity of enzymes involved in lactate turnorver (HK, PDH and CS) might be related to the time window of lactate perfusion (120 min), which was probably insufficient to induce transcription of lactate regulatory proteins, and consequently affecting the studied enzyme´s activity.

Even though increased PGC1α mRNA levels are considered an indicator of mitochondrial biogenesis, no changes were observed in COXIV mRNA levels or in mitochondrial DNA content of lactate perfused hearts. However, PGC1α is also a key regulator of both carbohydrate and lipid metabolisms inducing glucose and fatty acid oxidation [40, 41]. Indeed, it has been demonstrated that PGC1α increases lactate uptake by upregulating the expression of MCT1 [42]. Therefore, these data reinforce the role played by lactate as modulator of components of lactate oxidation complex.

One interesting finding of our study was that lactate perfusion reduced cardiac perfusion pressure with no additional impact on cardiac function. This finding corroborates findings of Montoya et al. [43], who demonstrated that lactate is a nitric oxide-dependent vasodilator increasing coronary perfusion. This is of particular interest since lactate-induced coronary vasodilation increases heart oxygenation improving its own metabolism.

Collectively, our data identifies lactate as an important modulator of genes involved in lactate oxidation complex in the cardiac tissue and this response seems to be mediated by an increase in NADH-induced O_2_^●-^/H_2_O_2_ production. These results provide new insights for the relative contribution of lactate probably driving its usage as energy fuel in conditions of physiological stress.

## Limitations

It is important to note that most data shown in the present study were obtained at 120 min of lactate perfusion of isolated hearts, since we could not sustain optimal conditions of cardiac function in latter time points. Therefore, our results are restricted to this time window. However, we cannot exclude distinct lactate signaling regulation of enzymes involved in lactate oxidation complex at different time points, including a possible increased enzyme´s activity at a later time. We also evaluated O_2_^●-^/H_2_O_2_ production mainly from non-mitochondrial sources, however we cannot totally exclude the participation of mitochondria in increasing O_2_^●-^/H_2_O_2_ levels derived by carbon oxidation.
